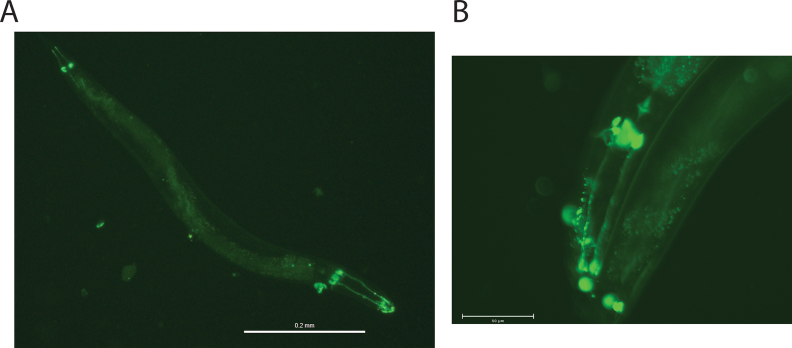# *C. elegans* model of riboflavin transporter deficiency (RTD) disorder reveals deficits in synaptic transmission and movement

**DOI:** 10.1016/j.gendis.2023.06.038

**Published:** 2023-08-08

**Authors:** Ramesh K. Narayanan, Gonzalo Perez-siles, Kamila A. Marzec, Alexandra Boyling, Brent Neumann, Manoj P. Menezes, Marina L. Kennerson

**Affiliations:** a Northcott Neuroscience Laboratory, ANZAC Research Institute - Sydney Local Health District, Sydney, NSW 2139, Australia; b Sydney Medical School, University of Sydney, Sydney, NSW 2050, Australia; c ANZAC Research Institute - Sydney Local Health District, Sydney, NSW 2139, Australia; d Neuroscience Program, Monash Biomedicine Discovery Institute and Department of Anatomy and Developmental Biology, Monash University, Melbourne, Victoria 3800, Australia; e TY Nelson Department of Neurology and Neurosurgery and Kids Neuroscience, Children's Hospital at Westmead, Westmead, Sydney, NSW 2145, Australia; f Paediatrics and Child Health, The University of Sydney, Sydney, Sydney, NSW 2145, Australia; g Molecular Medicine Laboratory, Concord General Repatriation Hospital, Sydney, NSW 2139, Australia

Riboflavin transporter deficiency (RTD), previously known as Brown-Vialetto–Van Laere syndrome, is a childhood-onset neurodegenerative disorder characterized by sensory and motor neuron degeneration causing ataxia, muscle weakness, optic atrophy, and respiratory failure. Mutations in *SLC52A2* and *SLC52A3*, solute carrier family members that encode riboflavin (RF) transporters RFVT2 and RFVT3, are known to cause RTD types 2 and 3, respectively.^1^ RF transport activity analysis showed that *SLC52A2* missense mutations caused a complete or moderate reduction in RF uptake and reduced RFVT2 protein expression *in vitro*,^1^ suggesting a loss-of-function disease mechanism.

RF/vitamin B2 is obtained from dietary sources and requires RF transporters for cellular uptake.^2^ RF is the precursor for flavin adenine dinucleotide and flavin mononucleotide, which are essential for mitochondrial bioenergetics as well as amino acid and fatty acid metabolism.^2^ Induced pluripotent stem cells (iPSCs) derived from RTD patients carrying the *SLC52A2* mutation showed significant structural and mitochondrial number abnormalities, abnormal lipid metabolism, and altered redox status.^2^ Patient motor neurons derived from iPSCs recapitulated the mitochondrial abnormalities observed in patient iPSCs and displayed significant changes in neuron morphology and impaired synaptic transmission.^2^^,^^3^ Interestingly, supplementation of RF partially restored the cellular phenotype and redox status of patient iPSCs,^2^ and high-dose oral RF therapy in RTD2 patients showed significant biochemical and clinical improvements.^1^ *In vitro* cellular models have significantly improved our understanding of RTD. However, they lack an intact nervous system with long axons and support cells. Therefore, developing *in vivo* models is crucial for furthering our understanding of RTD pathogenesis. Further, the development of effective therapies relies on the development of *in vivo* models that faithfully mimic RTD pathomechanisms.

Developing mammalian *in vivo* models for RTD is challenging, as RF transport deficiency resulted in embryonic lethality in mice.^4^ To overcome this, we generated *in vivo* models of RTD using *Caenorhabditis elegans* (*C. elegans*). The orthologue of human *SLC52A2* in *C. elegans* is *rft-1*. The corresponding RFVT2 amino acid residues (p.Y305 and p.L339) in which RTD mutations were reported are conserved in *C. elegans rft-1* (Fig. S1A). CRISPR-Cas9 was used to knock in the amino acid changes at the corresponding amino acid position in *rft-1* and the genotype was confirmed using Sanger sequencing (Fig. S1B).

Two independent *C. elegans* lines carrying the p.Y290C (homozygous) and p.L324P (homozygous) genotypes corresponding to the p.Y305C and p.L339P mutations, respectively, in *SLC52A2* were generated (Fig. 1A). The p.L324P mutation led to embryonic lethality in *C. elegans*, while the p.Y290C *rft-1* mutants were embryonically viable and were designated as *rft-1*^*Y290C*^ animals in this study. We measured the body width of *C. elegans*, which is used as a proxy for normal growth because cell size, rather than cell number, governs growth in this species. Interestingly, *rft-1*^*Y290C*^ animals displayed a significant reduction in mean body width compared with controls [control: 0.08466 ± 0.0008571 mm; *rft-1*^*Y290C*^: 0.07765 ± 0.0008093 mm] (Fig. 1B).

**Figure 1:**
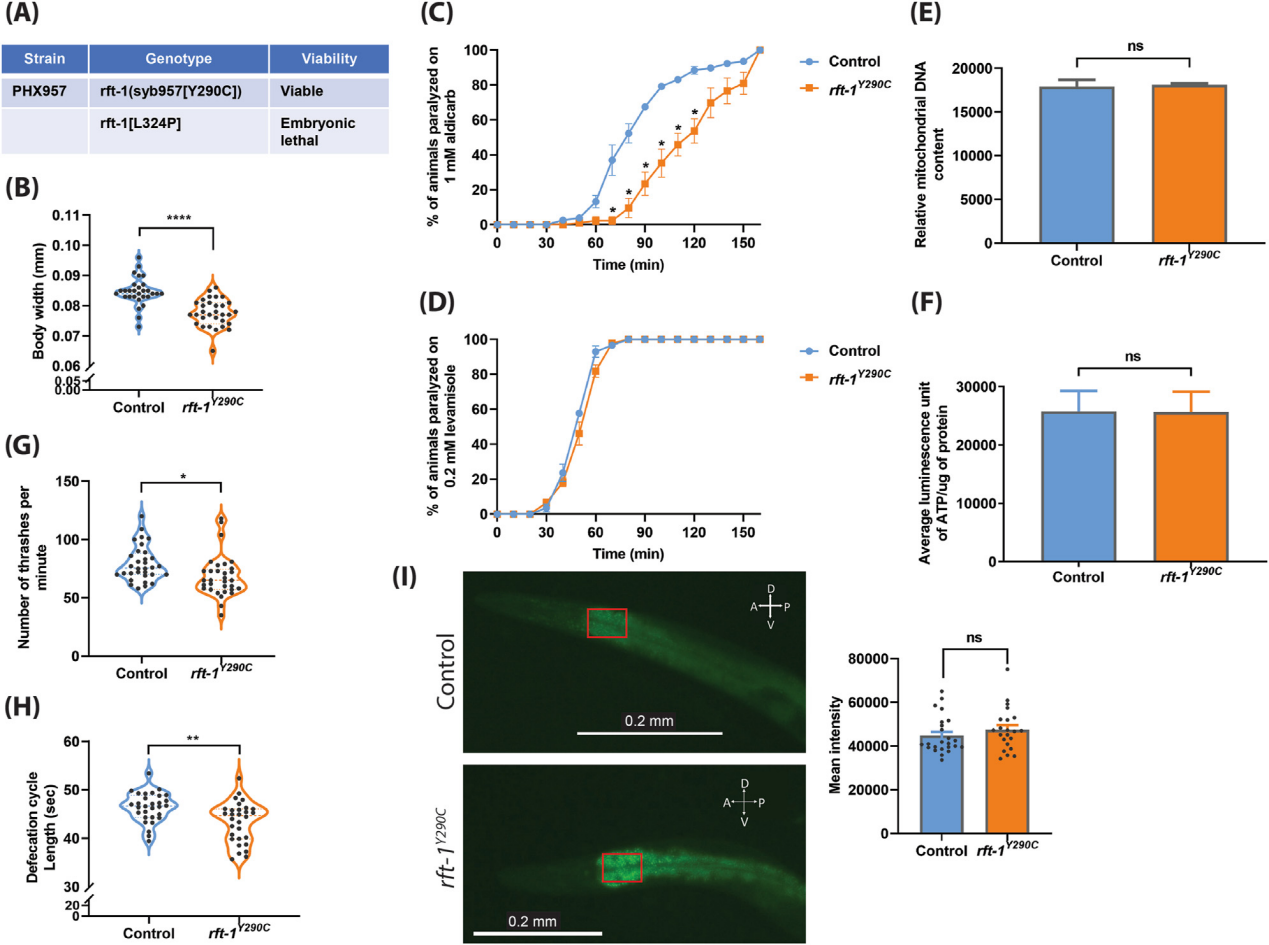
*Caenorhabditis elegans* as a model for studying functional and behavioral consequences of RTD-causing mutations in the riboflavin transporter gene *SLC52A2* (RFVT2). **(A)** The RTD strains generated and their viability. Knocking in the p.L324P mutation corresponding to the p.L339P mutation in human *SLC52A2* resulted in embryonic lethality and was, therefore, not characterized further. The p.Y290C mutation corresponding to the human p.Y305C *SLC52A2* mutation resulted in viable animals. **(B)** RTD animals show reduced body width. The number of animals used for body width measurements: EG7941 (*n* = 29) and *rft-1*^*Y290C*^ (*n* = 31). ^∗∗∗∗^Adjusted *P* < 0.0001, two-tailed unpaired *t*-test, Welch's correction. **(C, D)** Knocking in the human p.Y305C mutation into *Caenorhabditis elegans* riboflavin transporter gene *rft-1* causes synaptic transmission deficits. Aldicarb (an acetylcholine esterase inhibitor) and levamisole (an acetylcholine receptor antagonist) cause time-dependent paralysis in *Caenorhabditis elegans* and are widely used for identifying synaptic transmission mutants. *rft-1*^*Y290C*^ mutants displayed resistance to aldicarb when compared with control animals, suggesting synaptic transmission deficits associated with RTD mutation (C). The rate of paralysis of *rft-1*^*Y290C*^ animals is very similar to control animals in the presence of levamisole (D), indicating that the aldicarb resistance phenotype is due to pre-synaptic (neuronal) deficits. Twenty to thirty animals were used per replicate per genotype for aldicarb and levamisole analysis, and four replicates were used for each genotype. The data were presented as mean ± SE. ^∗^Adjusted *P* < 0.05, two-tailed unpaired *t*-test, Welch's correction. **(E, F)** Mitochondrial number and bioenergetics are not altered in *rft-1*^*Y290C*^ animals. PCR-based quantification of mitochondrial DNA was used as a proxy for mitochondrial content. *rft-1*^*Y290C*^ animals showed no change in mitochondrial number when compared with controls (E). Six one-day-old adult *rft-1*^*Y290C*^ animals were used per genotype per replicate for PCR-based quantification of mitochondrial DNA, and three replicates were performed for each genotype. Energy production in one-day-old adult *rft-1*^*Y290C*^ animals is shown in (F). ATP levels were unchanged in *rft-1*^*Y290C*^ animals when compared with wild-type controls. 1500 to 2000 one-day-old adult *rft-1*^*Y290C*^ animals were used per genotype per replicate for ATP quantification, and three replicates were performed for each genotype. Two-tailed unpaired *t*-test, Welch's correction; ns, not significant. **(G)** RTD animals display locomotion deficits. Four-day-old adult *rft-1*^*Y290C*^ animals displayed significant reductions in the number of body thrashes when compared with control animals (*n* = 32 for each genotype). ^∗^Adjusted *P* = 0.01, two-tailed unpaired *t*-test, Welch's correction. **(H)** Quantification of defecation cycle length in RTD animals. *rft-1*^*Y290C*^ animals exhibited reduced defecation cycle length when compared with EG7941 animals. Thirty animals were used per replicate per genotype for the defecation rate analysis. ^∗∗^Adjusted *P* = 0.0021, two-tailed unpaired *t*-test, Welch's correction. **(I)** Quantification of fat storage in *rft-1*^*Y290C*^ animals using RediStain™ WormDye Lipid Green (BODIPY stain) (which specifically labels lipid droplets, the fat storage organelle in *C. elegans*) reveals a trend for increased fat storage. However, the change in fat storage is not statistically significant. The area of the gut used for measuring lipid staining intensities was consistent for all the animals and is highlighted (red rectangle). Fiji (ImageJ) was used for measuring intensities which are represented in arbitrary units. The number of animals used for BODIPY staining are as follows: EG7941 (*n* = 24) and *rft-1*^*Y290C*^ (*n* = 22). Two-tailed unpaired *t*-test, Welch's correction; ns, not significant.

Nerve conduction and nerve biopsy studies showed that RTD2 presents with a neurodegenerative phenotype that has an axonal pathology^1^ and that motor neurons derived from the RTD-patient iPSCs displayed aberrant synaptic transmission.^5^ To assess synaptic transmission in *C. elegans*, chemical assays using aldicarb and levamisole are widely used. In the presence of aldicarb, an acetylcholinesterase inhibitor, *rft-1*^*Y290C*^ animals displayed a resistant phenotype indicating altered synaptic transmission. The time taken for 50% of the assayed animals to exhibit paralysis was 80 min for control animals and 120 min for *rft-1*^*Y290C*^ animals (Fig. 1C). However, in the presence of levamisole, an acetylcholine receptor antagonist, RTD animals behaved similarly to controls (Fig. 1D), suggesting that the aldicarb-resistant phenotype of *rft-1*^*Y290C*^ animals is mainly due to axonal defects and does not involve muscle. Our RTD mutants displayed aberrant synaptic transmission, similar to patient iPSC-derived motor neurons.^5^ Our data suggest that axonal defects contribute to the aberrant synaptic transmission in *rft-1*^*Y290C*^ animals, which recapitulates the axonal phenotype observed in RTD patients.

RTD iPSCs and iPSC-derived motor neurons displayed significant abnormalities in mitochondrial morphology and bioenergetics.^2^ Depletion of *rft-1* in *C. elegans* resulted in disrupted mitochondrial respiration and reduced ATP production.^4^ However, mitochondrial number and ATP levels were normal in our *rft-1*^*Y290C*^ animals (Fig. 1E, F). When compared with controls, the locomotion analysis of *rft-1*^*Y290C*^ animals showed a slight yet significant reduction (∼13%) in the number of body thrashes (Fig. 1G). We then investigated the motor neuron morphology of *rft-1*^*Y290C*^ animals by crossing RTD mutants with a *C. elegans* strain that expresses GFP in motor neurons responsible for locomotion in *C. elegans* [QH3659 - *ynIs37 (Pflp-13::GFP)*]. The motor neuron morphology of *rft-1*^*Y290C*^ animals was normal (Fig. S2). Since RTD is a sensory-motor disorder, we further analyzed the neuron morphology of amphid sensory neurons in *C. elegans* using a lipophilic dye (Fig. S3). Preliminary analysis showed that amphid sensory neurons were also normal in *rft-1*^*Y290C*^ mutants.

*C. elegans* neuronal gene expression (CeNGEN) data suggests that *rft-1* is highly expressed in the motor neuron (AVL) responsible for defecation. To investigate the effect of the RTD-causing mutation on AVL neuron function, we assessed the defecation cycle time of the *rft-1*^*Y290C*^ animals. The defecation cycle length was significantly reduced in the RTD mutants (control: 46.41 ± 0.5521 s; *rft-1*^*Y290C*^: 43.37 ± 0.7628 s) (Fig. 1H). RF plays an important role in fatty acid beta oxidation, a process required for breaking down fatty acids to produce energy.^1^^,^^2^ RTD mutation reduces RF uptake by the RFVT2 transporter. Consequently, disrupted fatty acid beta oxidation may lead to increased fat storage, similar to the lipid metabolism abnormalities observed in patients and patient-derived iPSCs.^1^^,^^2^ Knockdown of *rft-1 in vivo* has been reported to increase fat storage in *C. elegans*.^4^ We investigated whether *rft-1*^*Y290C*^ mutants displayed abnormal fat storage compared with control animals using the BODIPY stain to label fat storage organelles. *rft-1*^*Y290C*^ mutants displayed a slight increase in fat storage, but this was not statistically significant (Fig. 1I).

To conclude, we generated a knock-in *C. elegans* model of RTD disorder that recapitulates some of the clinical features of RTD. We demonstrated that the *C. elegans* neuronal RF transporter played a significant role in synaptic transmission. Our data indicate that the RTD-causing mutation disrupts this process, thus affecting neuron health and function, which may result in the axonal defect observed in RTD patients. However, our *C. elegans* model did not replicate the ATP deficits observed in patient-derived *in vitro* cell models. Like mammals, *C. elegans* have two RF transporters, *rft-1* and *rft-2*, which are predominantly expressed in neurons and intestinal cells, respectively. The *rft-1*^*Y290C*^ animals expressed fully functional *rft-2*, which might compensate for the partial loss of function caused by the RTD mutation in *rft-1*. Therefore, the effect of *rft-2* knockdown in *rft-1*^*Y290C*^ animals and its impact on energy production and fat metabolism requires further investigation. Our knock-in model complements the *C. elegans* knockdown model of RTD. Future comparative metabolomic and proteomic studies on these two *C. elegans* models will help further understand the molecular signatures of disease pathogenesis and facilitate the development of novel therapeutics for RTD.

## Author contributions

RKN: conceptualization, experimentation, analysis, writing - original draft, and writing - review & editing; GPS: ATP quantification assay; KAM: microscopy; AB: protein quantification; BN: experimental design and writing – review & editing; MPM and MLK: conceptualization, supervision, and writing - review & editing.

## Conflict of interests

All authors declare no conflict of interests.

## Funding

Financial support was provided by the Cure RTD Foundation and Australian Medical Research Future Fund (MRFF) Genomics Health Futures Mission Grant (No. 2007681).

## Figures and Tables

**figs1:**
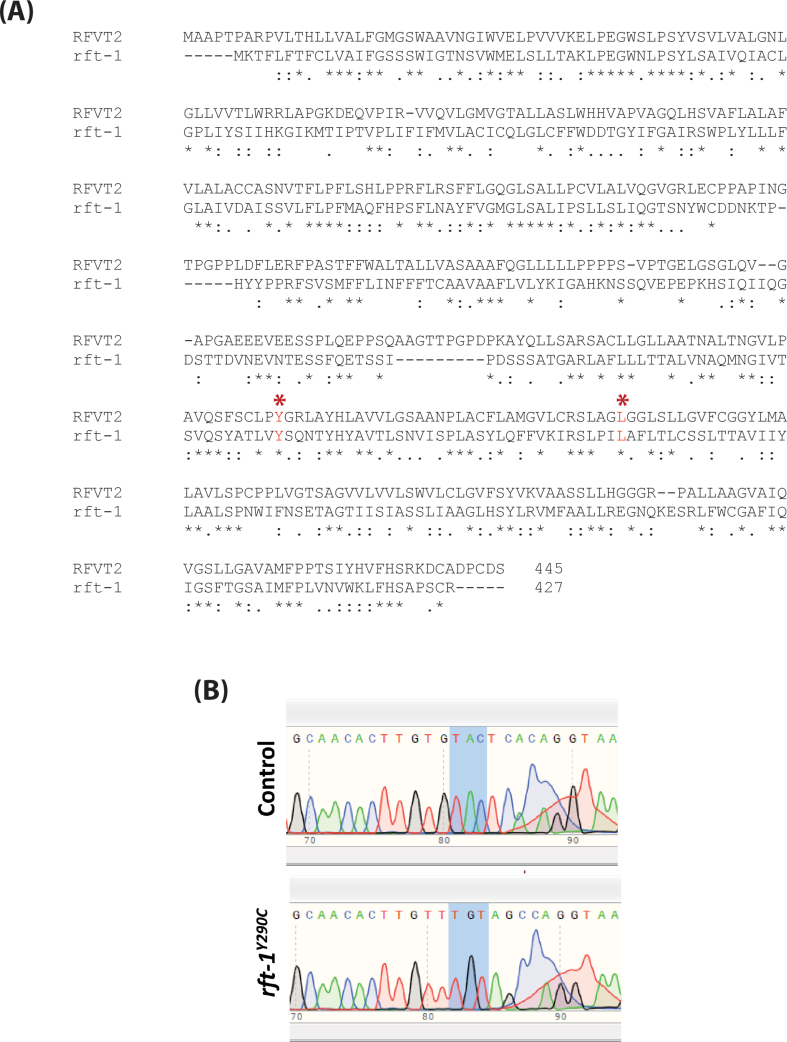


**figs2:**
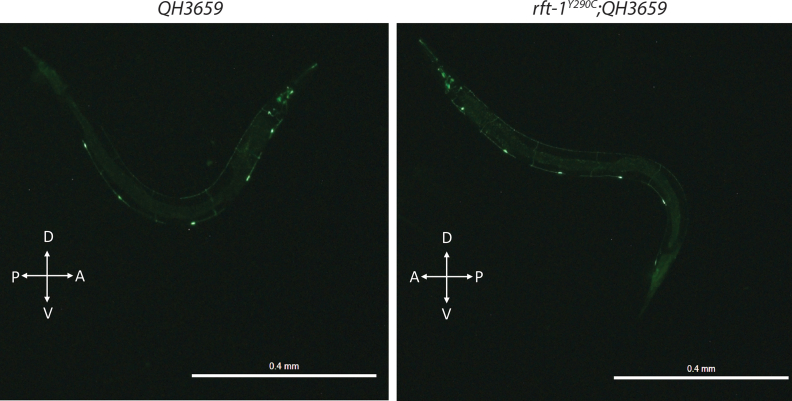


**figs3:**