# Supplementary material for: C. elegans model of riboflavin transporter deficiency (RTD) disorder reveals deficits in synaptic transmission and movement
Source: Genes Dis. 2023 Aug 8;11(4):101071. doi: 10.1016/j.gendis.2023.06.038 (PMC10955194; doi:10.1016/j.gendis.2023.06.038)
Supplement: Multimedia component 1 [file mmc1.zip › gendis_1071_HIGHLI_2 23-556_mmc1.DOC]

**Supplementary information**

**Supplementary Figure 1.** (A) Sequence alignment of RFVT2 (SLC52A2) and RFT-1 amino acid residues using clustalW. Conserved amino acid residues between RFT-1 and RFVT2 are highlighted by *. The conserved amino acid residues in *C. elegans* corresponding to the human RFVT2 residues harbouring RTD mutations are highlighted red asterisk *. (B) Sanger sequencing chromatogram of EG7941 and *rft-1^Y290C^* animals. Highlighted region shows the nucleotide change from TAC to TGT that changes the amino acid residue from tyrosine (Y) to cysteine (C) in *rft-1^Y290C^* animals.

**Supplementary Figure 2.** Motor neuron morphology is unaffected in RTD animals. Representative images of day 1-old imaging control QH3659 [*ynIs37(Pflp-13::GFP)*] and *rft-1^Y290C^* mutant carrying the *ynIs37* allele. Cell body, commissure, and axonal projections of GABAergic DD motor neurons in RTD mutants showed no signs of motor neuron degeneration. Age synchronized day 1-old animals were anesthetized using 100 mM levamisole and mounted on a 2% agar pad and imaged using a Leica DMI3000B inverted microscope and a ProgRes CF^Cool^ Camera.

**Supplementary Figure 3.** Representative images of amphid sensory neurons of EG7941 animals stained using a lipophilic dye RediStain™ WormDye Neuro Green Dio. (A) Head and tail amphid sensory neurons of an adult hermaphrodite imaged immediately after staining (B) Magnification of the head amphid sensory neurons for a different animal imaged using EVOS FL Auto 2 (40X; coverslip corrected).

**Methods**

**Animals**

*C. elegans* were maintained on nematode growth medium at 22°C as previously described ^1^. The EG7941 [unc-119 (ed3)III; oxTi396 [eft-3p::tdTomato::H2B::unc-54 3'UTR + Cbr-unc-119(+)]] strain was used as the background animal for introducing RTD mutations to generate *rft-1*(syb957[Y290C]); unc-119 (ed3)III; oxTi396 [eft-3p::tdTomato::H2B::unc-54 3'UTR + Cbr-unc-119(+)] animals. EG7941 animals were used as controls in our experiments. For genotyping experiments single worm PCR amplification was performed as previously described ^2^ using the following primers: Forward 5’-ATTCTAAAAATGCTGAAAAT-3′and Reverse 5’-ATTGTAGATACGATGCCAGT-3′. An exempt dealing approval was obtained from The University of Sydney Institutional Biosafety Committee (IBC) for this project.

**Body width measurement**

Animals (48 h post L4 stage) were used for the body width measurement experiment. Age-synchronized wildtype and RTD mutant animals were mounted on 3% (w/v) agar pads and anesthetized using 100 mM levamisole. Images were captured using a Leica DMI3000B inverted microscope and a ProgRes CF Cool Camera. Body width measurements were carried our as previously described ^2,3^.

**Body thrash count**

Day 4-old EG7941 and *rft-1^Y290C^* animals were utilized for body thrash assay. Thrashing assays were performed on 3 cm unseeded NGM plates containing 1 mL of M9 buffer as previously described ^2,4^. Following a minute acclimation period in M9 buffer, the movement of the animal’s head and tail to the same side was counted as a single body thrash and the number of thrashes per minute was reported.

**Defecation cycle length assay**

The defecation cycle length of EG7941 and *rft-1^Y290C^* animals was calculated by measuring the time interval between two consecutive pBoc contractions as previously described ^5^.

**Fat storage quantification**

Day 1-old animals were used for fat storage analysis. Lipid droplets, a major fat storage organelle in *C. elegans* was stained using RediStain™ WormDye Lipid Green (also known as BODIPY stain) according to manufacturer’s instructions. The area of gut used for quantification was consistent between all the animals and highlighted in Figure 1I.

**Neuron morphology**

QH3659 [*ynIs37(Pflp-13::GFP)*] animals express GFP in the six DD motor neurons that are involved in animal locomotion. *C. elegans* mutants were crossed with QH3659 animals to generate RTD animals that expresses GFP in DD neurons. Day 1-old animals were mounted on a 3% (w/v) agar containing 100 mM levamisole and imaged with a Leica DMI3000B inverted microscope and ProgRes CF^Cool^ Camera. The images were taken using 10X objective and the exposure time was 250 ms. Amphid sensory neurons were stained using a lipophilic dye RediStain™ WormDye Neuro Green Dio according to manufacturer’s instructions.

Chemical assays (Aldicarb and levamisole assay) for screening synaptic transmission mutants and the mitochondrial DNA copy number PCR and ATP quantification experiments were performed as previously described ^2^.

**Statistical analysis**

Prism software was used for statistical analysis. Two-tailed unpaired t-test and Welch’s correction was used to calculate adjusted p-values in this study. P-value < 0.05 was regarded as significant.

References ^6-13^ are additional literature that informed our study design but were not included in the main body of the article.

**Reference**

1. Brenner S. The genetics of Caenorhabditis elegans. Genetics 1974;77:71-94.

2. Narayanan RK, Brewer MH, Perez-Siles G, et al. Charcot-Marie-tooth disease causing mutation (p.R158H) in pyruvate dehydrogenase kinase 3 (PDK3) affects synaptic transmission, ATP production and causes neurodegeneration in a CMTX6 C. elegans model. Hum Mol Genet 2021;31:133-45.

3. Morck C, Pilon M. C. elegans feeding defective mutants have shorter body lengths and increased autophagy. BMC Dev Biol 2006;6:39.

4. Nawa M, Kage-Nakadai E, Aiso S, Okamoto K, Mitani S, Matsuoka M. Reduced expression of BTBD10, an Akt activator, leads to motor neuron death. Cell Death Differ 2012;19:1398-407.

5. Cooper JF, Dues DJ, Spielbauer KK, Machiela E, Senchuk MM, Van Raamsdonk JM. Delaying aging is neuroprotective in Parkinson's disease: a genetic analysis in C. elegans models. NPJ Parkinsons Dis 2015;1:15022.

6. Biswas A, Elmatari D, Rothman J, LaMunyon CW, Said HM. Identification and Functional Characterization of the Caenorhabditis elegans Riboflavin Transporters rft-1 and rft-2. Plos One 2013;8.

7. Rizzo F, Ramirez A, Compagnucci C, et al. Genome-wide RNA-seq of iPSC-derived motor neurons indicates selective cytoskeletal perturbation in Brown-Vialetto disease that is partially rescued by riboflavin. Sci Rep-Uk 2017;7.

8. Udhayabanu T, Manole A, Rajeshwari M, Varalakshmi P, Houlden H, Ashokkumar B. Riboflavin Responsive Mitochondrial Dysfunction in Neurodegenerative Diseases. J Clin Med 2017;6.

9. Colasuonno F, Bertini E, Tartaglia M, Compagnucci C, Moreno S. Mitochondrial Abnormalities in Induced Pluripotent Stem Cells-Derived Motor Neurons from Patients with Riboflavin Transporter Deficiency. Antioxidants-Basel 2020;9.

10. Marioli C, Magliocca V, Petrini S, et al. Antioxidant Amelioration of Riboflavin Transporter Deficiency in Motoneurons Derived from Patient-Specific Induced Pluripotent Stem Cells. Int J Mol Sci 2020;21.

11. Balasubramaniam S, Christodoulou J, Rahman S. Disorders of riboflavin metabolism. J Inherit Metab Dis 2019;42:608-19.

12. Manole A, Jaunmuktane Z, Hargreaves I, et al. Clinical, pathological and functional characterization of riboflavin-responsive neuropathy. Brain 2017;140:2820-37.

13. Taylor SR, Santpere G, Weinreb A, et al. Molecular topography of an entire nervous system. Cell 2021;184:4329-47 e23.
